# Brain Activity Associated with the Planning Process during the Long-Time Learning of the Tower of Hanoi (ToH) Task: A Pilot Study

**DOI:** 10.3390/s22218283

**Published:** 2022-10-28

**Authors:** Keita Mitani, Namal Rathnayake, Upaka Rathnayake, Tuan Linh Dang, Yukinobu Hoshino

**Affiliations:** 1 School of Systems Engineering, Kochi University of Technology, 185 Miyanokuchi, Tosayamada, Kami 782-8502, Kochi, Japan; 2 Department of Civil Engineering, Faculty of Engineering, Sri Lanka Institute of Information Technology, Malabe 10115, Sri Lanka; 3 School of Information and Communications Technology, Hanoi University of Science and Technology, No. 1 Dai Co Viet Road, Hanoi 100000, Vietnam

**Keywords:** long-time learning, learning curve, Tower of Hanoi, executive function, planning, planning and decision-making

## Abstract

Planning and decision-making are critical managerial functions involving the brain’s executive functions. However, little is known about the effect of cerebral activity during long-time learning while planning and decision-making. This study investigated the impact of planning and decision-making processes in long-time learning, focusing on a cerebral activity before and after learning. The methodology of this study involves the Tower of Hanoi (ToH) to investigate executive functions related to the learning process. Generally, ToH is used to measure baseline performance, learning rate, offline learning (following overnight retention), and transfer. However, this study performs experiments on long-time learning effects for ToH solving. The participants were involved in learning the task over seven weeks. Learning progress was evaluated based on improvement in performance and correlations with the learning curve. All participants showed a significant improvement in planning and decision-making over seven weeks of time duration. Brain activation results from fMRI showed a statistically significant decrease in the activation degree in the dorsolateral prefrontal cortex, parietal lobe, inferior frontal gyrus, and premotor cortex between before and after learning. Our pilot study showed that updating information and shifting issue rules were found in the frontal lobe. Through monitoring performance, we can describe the effect of long-time learning initiated at the frontal lobe and then convert it to a task execution function by analyzing the frontal lobe maps. This process can be observed by comparing the learning curve and the fMRI maps. It was also clear that the degree of activation tends to decrease with the number of tasks, such as through the mid-phase and the end-phase of training. The elucidation of this structure is closely related to decision-making in human behavior, where brain dynamics differ between “thinking and behavior” during complex thinking in the early stages of training and instantaneous “thinking and behavior” after sufficient training. Since this is related to human learning, elucidating these mechanisms will allow the construction of a brain function map model that can be used universally for all training tasks.

## 1. Introduction

Identifying the areas of significant activation in the brain and their relation to executive function, including cognition, as a particular brain mechanism [1], is essential to clarify the effects to investigate the impact of planning and decision-making processes in long-time learning. Executive function is defined as goal–direction behavior, including planning for the short-term future. The ability to maintain an appropriate attitude for achieving future goals relies on four main points, according to Lezak et al., such as goal formulation, planning, carrying out goal-directed plans, and effective performance [2]. Executive function involves higher-order information from any sensory stimulus. Higher-order executive function processes transmit information to the brain. The prefrontal cortex (PFC) is closely involved in executive function and plays crucial roles in planning, executive processing, and emotional expression. The Tower of Hanoi (ToH) task measures planning abilities by systematically varying planning demands [3,4,5].

In the ToH task, a player must rearrange a set of multiple disks on three pegs of varying sizes from the start state to the goal state using the minimum number of moves. Completing this task is known to be critically dependent on PFC activation [1,6,7,8,9,10,11,12,13]. Therefore, the ToH task is considered appropriate for investigating the role of the PFC.

On the other hand, previous studies have confirmed activation of the dorsolateral PFC (DLPFC) during the ToH task. Some researchers have argued that the ToH task activates the DLPFC (BA 9), parietal lobe (BA 7, 40), inferior frontal gyrus (BA 44), and premotor cortex (BA 6) [14,15,16,17,18].

Regarding learning, memories and expressions are established by biological feedback, such as visual perception and haptics. This feedback affects the learning processing time and efficiency. The executive function consists of the same lower-level functions, such as divided attention, processing ability for multiple tasks, conversion ability of a thought set, thinking speed, and inductive guessing [2].

Impaired executive function, which significantly influences individual ability, planning, prioritization, organization, attention and detailed memory, emotional response control, and planning and decision-making [19], is considered to include the frontal lobe. Executive function and planning require foresight and empirical knowledge. Planning and decision-making become logical choices from available options. In other words, this can explain the evaluation and selection of several conflicting alternatives.

When making a good choice, it is necessary to measure the advantages and disadvantages of all considerations. For effective planning and decision-making, predicting each option’s outcome from a task operation is essential. The best item from all option items depends on the outcome predicted for the whole situation. Therefore, all planning and decision-making result from a definitive choice [20].

Interactions in a task environment are integrated into ongoing procedures/proceedings and are significant in the planning and decision-making process from a recognition perspective. The planning and decision-making results are related to an unchangeable selection based on logic and rationality.

Card et al. studied the minimum reaction time when starting an exercise after a short-time judgment of human acquisition from visual information. The model human processor (MHP) is a famous model of human processing developed by Card [21].

Humans have a minimal response time of more than 370 ms (eye movement processor = 230 ms, cognitive processor = 70 ms, motor processor = 70 ms) from the time information is sent and the planning and decision behavior. Puzzle tasks in this experiment do not have any delay or prediction error between the screen information and behavior. By pressing a button, participants can predict the next state based on planning and decision-making.

This planning and decision-making do not include an element of probability. The operation depends on precise planning and decision, and participants can perform the task in less time. The time lags during these operations are caused mainly by the planning and decision-making process based on visual information. In some control cases for various planning and decision-making processes, the time lag is about 400–500 ms [21]. If learning levels are sufficiently advanced, participants can control these times. Trends in the time lags in button pressing correlate with trends in the temporal learning time. Functional magnetic resonance imaging (fMRI) and outside training environments have the same time lag and learning properties.

The present study focuses on the brain regions mainly involved in executive function, including the DLPFC, parietal lobe, inferior frontal gyrus, and premotor cortex [14,15,16,17,18]. Our investigation is to observe changes in the degree of activation depending on the learning status of the learners.

This long-time learning experiment was conducted using a ToH puzzle, which is frequently used as a task to measure learning ability [1,22,23,24,25]. The long-time learning experiment consisted of a learning term and three MRI terms. In the learning term, learners studied the ToH puzzle outside the fMRI scanner for seven weeks. The three MRI terms were conducted at the end of the learning term’s starting, middle, and final third.

## 2. The Tower of Hanoi (ToH)

The ToH is a popular puzzle in cognitive psychology and neuropsychology used to assess a set of behaviors collectively referred to as executive function. In addition, the ToH is also a popular puzzle game for cognitive science and neuroscience [1,22,23,24,25]. This task is typically used to evaluate behaviors and executive function. In the current experiment, estimating the brain activation recursively was attempted. Inductive inference is an estimation method that identifies general rules of individual, partial, and special events [26,27].

The ToH puzzle is a complex cognitive task in which participants must learn the procedural process for disk operation. The guidelines of this game can be explained as follows.

**Figure 1:**
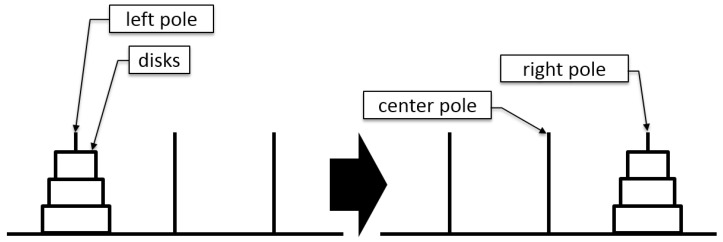
The ToH experimental task.

The detailed rules are as follows:

1. The ToH puzzle consists of three poles and different-sized disks.
2. In the initial position, all disks are stacked on the left pole in ascending order.
3. Participants can only move one disk at a time.
4. The disks can be moved from one pole to another.
5. The disks can be placed on an empty pole or larger disks.
6. A larger disk cannot be placed on top of a smaller disk.
7. The goal is to move all the disks to the right pole, as shown in Figure 1.
8. For *n* disks, the optimal solution path is $2^{n-1}$ moves.
9. The game ends when all disks are moved to the right pole.

The new hypothesis consists of the procedure of trial and error. Players acquire particular strategies by trial and error method in the ToH puzzle. These trials contribute to the final solution to reproduce the completed moving procedure. The optimal number of movements for the three disks in the ToH puzzle is seven; however, if the frequency of the trial and error decreases, the thinking process regarding executive function shifts to a working memory task instead. This process depends on the ability of the executive function.

The planning ability is enhanced by the trial and error method. In this case, the planning time would be longer than that for working memory. The brain regions associated with executive function activate if a player is making plans for a long time.

After that, this process would shift to a working memory task. Due to the complexity of the task, players cannot memorize the whole process. Players can memorize patchy rules of the solving process. Hence, those disjointed rules act in wide-area memory regions.

In the main experiment, participants learned the ToH task for seven weeks. The frequencies of button presses and achievements were recorded for each participant. Difficulty levels can be modified according to game times and the number of disks. In our analysis, the repeat task was prepared as a control task. In repeat tasks, participants push a button and do not play the game. Brain activities during the repeat task are independent of planning and decision-making. At the same time, the participant watches a video of a previous ToH task. This task has the same visual stimulus as the ToH task.

Moreover, participants perform these tasks without using executive functions. Thus, this repeat task is performed only by pushing a button and not playing the ToH. It is a type of dummy operation. In this case, particular responses appear in the motor and visual cortices. This analysis method uses two types of fMRI scans.

The first one is the scan of the ToH task. These data involve the use of the executive function. The other is a scan of the repeat task image data without the executive function. Calculating differences between tasks and repeats can check the activation of executive functions.

## 3. Experimental Work

Before the experiment, the participants were interviewed about their knowledge and experiences with the ToH puzzle. All participants had some basic knowledge about the ToH puzzle. Player knowledge of the ToH was the same for the experimental baseline. Therefore, this knowledge can be defined as a social composition condition.

**Figure 2:**
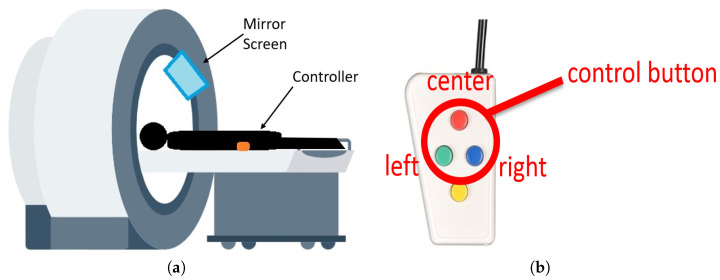
fMRI machine setup used in the experiments [28]. (**a**) Mirror screen in the fMRI machine; (**b**) Controller used in the experiment while in the fMRI machine.

A screen located in front of the participant showed the ToH task. The participant solves a five-disk ToH puzzle in the experiment. The task is to play the ToH using a four-button controller, as shown in Figure 2b. Participants can attempt to solve the puzzle on a screen using a controller, as shown in Figure 2a. The participant lies inside the fMRI to play the ToH puzzle task while holding a controller in the right hand. The controller has four buttons, and this experiment uses the left, top, and right buttons corresponding to the left, center, and right poles in the ToH.

**Figure 3:**
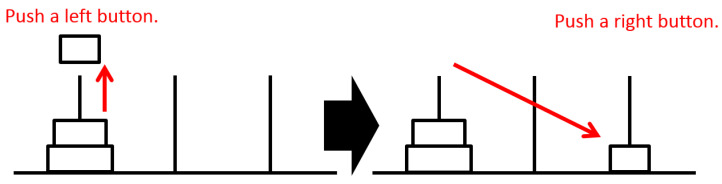
How to control the disks in the ToH.

Participants can press a button corresponding to a pole on which disks are stacked. When the button is pressed, the disk at the corresponding pole is lifted, as shown on the left in Figure 3. Next, the participant can press a button corresponding to a target pole. The lifted disk moves to the target pole at that time, as shown on the right in Figure 3. The task is considered complete after all disks are on the right pole. Then, the disks are reset to the left pole as the initial position for the next game. Participants repeated the ToH task continuously within a given time.

**Figure 4:**
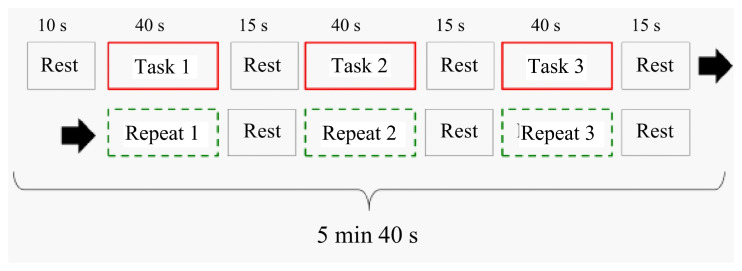
Task sequence during one session.

Figure 4 shows one session of the MRI experimental sequence. This experiment consisted of three tasks and three repeats. In the Task 1 period, the task started from the initial state. The purpose was to confirm the activation associated with executive function based on activation differences between task and repeat scans. All disks were set on the left pole as the initial position. The disk starting position in Task 2 was from the ending position in Task 1. The disk starting position in Task 3 was from the ending position in Task 2. Tasks 1, 2, and 3 only differed regarding the disk starting position. It repeated 1, 2, and 3, and a video of Tasks 1, 2, and 3 was shown. All task and repeat times were 40 s. The rest time between tasks was 10 s, and the rest time between tasks and repeats was 15 s. The total time of one session was 5 min and 40 s (136 images were scanned).

During the task period, the participant played the ToH. Participants continuously played as many times as instructed. During the task 1 period, the task started from the initial state. All disks were set on the left pole as the initial position. During the Task 2 period, the participants continued solving the puzzle from the final state of the previous task.

During the repeat period, the participants synchronously pressed the button while watching the video recorded during the task period. During this period, the push button did not affect the time performance or executive function tasks. Therefore, images during this period included only activation information in the motor and visual cortices based on controller operations. They did not have brain activation information regarding executive function, and the experiment was intended to extract only activation information. This image differed between the task and repeat blocks.

During the rest period, a fixation cross was displayed for the participant to focus. The color of the fixation cross depended on the next period. If the task period was next, the fixation cross color was red. If the next was the repeat period, then the color of the fixation cross was light blue.

The measurement method utilizes the magnetic resonance scanning method of brain activity. One scan can be performed within 3 s. Scan voxels at the start of brain activity are not included in the analysis because they are unstable as brain activity. They are imaged at a voxel resolution of 1–8 mm${}^{3}$.

In data analysis, realign (motion correction), normalize (standardization), smooth (smoothing), specify-1st-level-modeling (standard brain model determination), and estimate (brain activity grayscale images are created in the flow of the estimated activity region). By taking the difference between them, the active part is determined. Active areas are narrowed down to *p* > 0.001 significant active areas by *t*-test.

## 4. The Long-Time Learning Experiment Environment

The long-time learning experiment was conducted with four participants. These experiments need a longer time to process the scanning of 402 image data samples. Many people believe continuous scanning is a health risk. Therefore, finding volunteers for these types of studies is difficult. In addition, the scanning process is rather expensive. Considering all of these concerns, only four samples were tested for this experiment. However, to have a uniform study, the participants were chosen at similar ages (21–23 years old). In addition, both males and females were considered for these experiments. The attributes of the participants are as follows. To reduce the complications and provide a repeatable experimental background, the female participants were examined in the Ovulation phase of the menstrual cycle.

- Participant 1 was male, 21 years old, and right-handed.
- Participant 2 was male, 23 years old, and right-handed.
- Participant 3 was female, 21 years old, and right-handed.
- Participant 4 was male, 21 years old, and right-handed.

**Figure 5:**
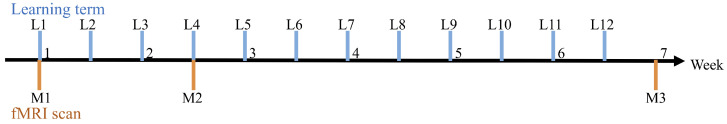
Experimental flow over 7 weeks.

Figure 5 shows the experimental flow. This learning experiment was conducted for seven weeks. The participants learned to solve the ToH for 40 min once or twice a week outside the MRI. Participants 1 and 2 participated in 12 learning sessions (L1–L12 in Figure 5). Participants 3 and 4 participated in a total of 10 learning sessions. Though the number of sessions used to train the participants differed (10 and 12), the evaluation was done constantly. Therefore, overlearning by participants 1 and 2 can be disregarded.

To observe the participants’ brain activity and progress in and convergence of learning, they underwent fMRI scans three times while solving the ToH (M1–M3 in Figure 5) in this experiment. fMRI scans were performed immediately after the first (L1), fourth (L4), and last learning periods (L12 or L10).

The interval between M1–M2 was ten days, and the interval between M2–M3 was 30 days. This time delay was considered due to the health risk of continuous scanning of MRI. Generally, successive MRI scans for a more extended period are prohibited due to the strong magnetic field effect of the machine. Therefore, the scanning time was limited to no longer than 30 min with an interval of more than 7–10 days.

This experiment used a MAGNETOM Verio 3T scanner (SIEMENS Co., Ltd., Erlangen, Germany) for acquiring fMRI images. Head movement was limited with the use of mild restraints and cushioning. The imaging parameters were TR = 2500 ms, TE = 30 ms, FoV = 192 mm${}^{2}$, voxel size = 3.0 mm${}^{3}$, and slice thickness = 3.0 mm. 136 scanned for analysis images per session. Two images were excluded from the analysis because the longitudinal magnetization of the tissue was unsteady.

Furthermore, an anatomical image with a resolution of 1.0 mm${}^{3}$ was combined with a T1-weighted image to obtain positional information. A projector was installed outside the MRI room and projected the experimental task image on a resin screen near the head-side opening of the fMRI device through a telephoto lens. The participants watched the images on the screen via a mirror placed over the coil above their heads.

In this experiment, the participants performed a ToH task involving five disks. Participants lying on the bed inside the fMRI machine performed the ToH task using a controller in their right hand. Participants repeatedly carried out the ToH task within a given time. The experimental design employed was a block design with alternating tasks and rest periods. Performance data during the experiment were recorded to confirm the learning progress.

Moreover, fMRI images were obtained for each learning process. The five-disk ToH task was performed inside the fMRI machine, and the time allotted for each task was 40 s. Three ToH tasks using five disks are blocks within a certain period.

### Software Platform of Experiments

SPM12 software (Wellcome Centre for Human Neuroimaging, London, UK) was used to process and analyze the fMRI data. A total of 408 images (136 images for each M1, M2, and M3 instance) were obtained from three fMRI-runs. The first two scan images were discarded from each fMRI- run. Thus, this analysis used 402 scan images (408−(2×3) instances).

These first two scan images were discarded because the magnetization of the MRI was not in a steady state at the beginning of each scan. Functional images were corrected for differences in slice acquisition time and motion artifacts.

This analysis examined the degree of activation between the early and late stages of learning at each region of interest (ROI). The data were realigned, normalized according to the standard Montreal Neurological Institute (MNI) model, and smoothed with an 8-mm full-width–half-width Gaussian filter. MNI coordinates were used for the brain activation analysis. Only focused voxels were analyzed using the WFU PickAtlas toolbox for masking [29,30].

## 5. Results

The degree of progress was the learning level indicator for all participants. The calculation method for the degree of progress is as follows.

**Figure 6:**
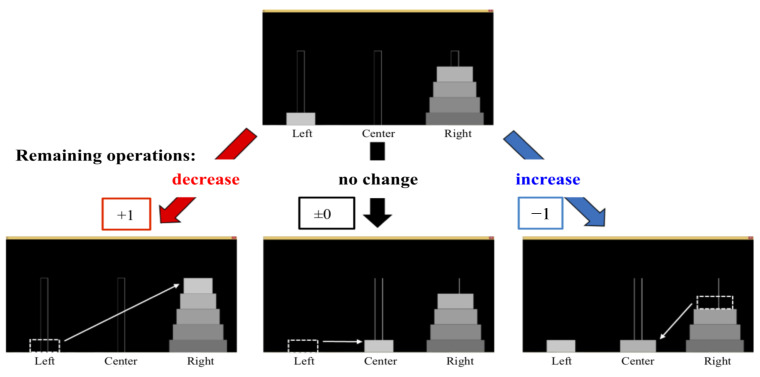
The evaluation method for the degree of progress.

The first step was to find the shortest number of moves remaining on all the boards. The minimum number of moves for the 5 disks was 31 from the initial position, and the minimum number of remaining movements is less than 31 moves from the other starting position. One point was added to the degree of progress when the remaining operations decreased by one step. If there was no change in the remaining operation, the degree of improvement remained the same. Conversely, one point was subtracted from the degree of progress when the remaining operations increased by one step. Therefore, the total possible score when solving a puzzle was 31 points. The remaining operation was assumed to be a measurement of the learning level. The point calculations are shown in Figure 6.

Card’s MHP defines the fastest time a person can press the button as approximately 0.3 s [21]. In this case, since the time for one task was 40 s, the maximum number of evaluation points was about 130 (=40/0.3). If there are few remaining operations in the task, the degree of progress is a high score. In this case, a high score indicates a quick and accurate operation. Therefore, in this case, the learning level of the participant is high. The degree of progress is calculated to evaluate the learning level for the task.

**Figure 7:**
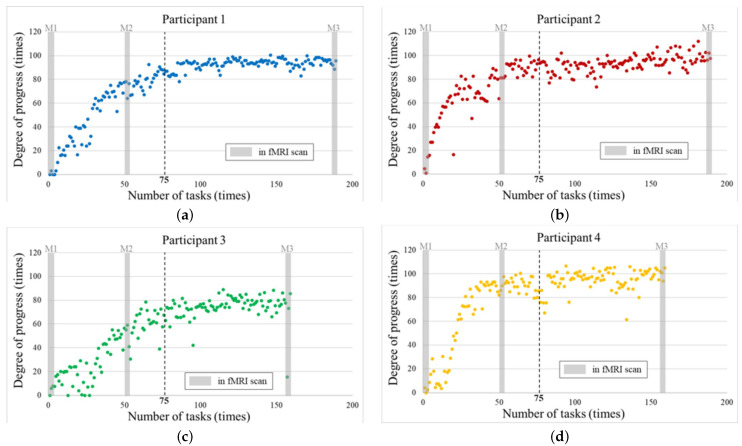
Learning curve for the degree of progress; (**a**) Learning curve for Participant 1; (**b**) Learning curve for Participant 2; (**c**) Learning curve for Participant 3; (**d**) Learning curve for Participant 4.

The participants completed the tasks between 150 and 180 times and underwent fMRI scans nine times. The degree of progress was calculated for each job. Figure 7 shows the learning curves for the degree of improvement for each participant. Their transitions can be confirmed based on the learning curves.

The learning curves converged after Task 75 (in L5). The gray part shows corresponding marks about the data from the fMRI scans (M1–M3). The observed learning progress for M1 and M3 are at the beginning and end areas of the learning period, respectively. Moreover, their learning was progressing in M2.

Four ROI masks were used for the DLPFC, parietal lobe, inferior frontal gyrus, and premotor areas. Several examples show an increasing or decreasing trend in brain activity as learning progresses [31,32]. There is a close relation to the learning behavior by brain regions that show such increasing/decreasing trends. In the first analysis, we observed differences in brain activity between the whole brain’s first and third fMRI scans. We searched for brain regions (voxels) that showed significant differences. We examined the relationship between learning progression and the activity level of each scan for the regions that showed significant differences in brain activity. We designed the contrasts and analyzed the brain activity on the SPM to verify this significant difference. The contrasts were “first fMRI scan vs. third fMRI scan” and “third fMRI scan vs. first fMRI scan”. These fMRI scans consisted of all tasks vs. all repeats in the same session. Statistical thresholds were set for uncorrected brain peak levels (p<0.001) and corrected cluster levels (p<0.05). For motion correction, all models included the six-dimension head-motion parameters as the regressor.

Executive function is critically dependent on PFC activation [1,6,7,8,9,10,11,12,13]. The PFC is believed to be involved in planning ability [19,33,34].

## 6. Discussion

Previous studies have confirmed the activation of the DLPFC (BA 9) in the ToH task. On the other hand, some researchers have argued for the activation of the parietal lobe (BA 7, 40), inferior frontal gyrus (BA 44), and premotor cortex (BA 6) [14,15,16,17,18]. These studies suggest that these brain regions are involved in planning ability. Investigations of these brain regions focus on the transition of brain activity at the peak coordinates in each task.

**Figure 8:**
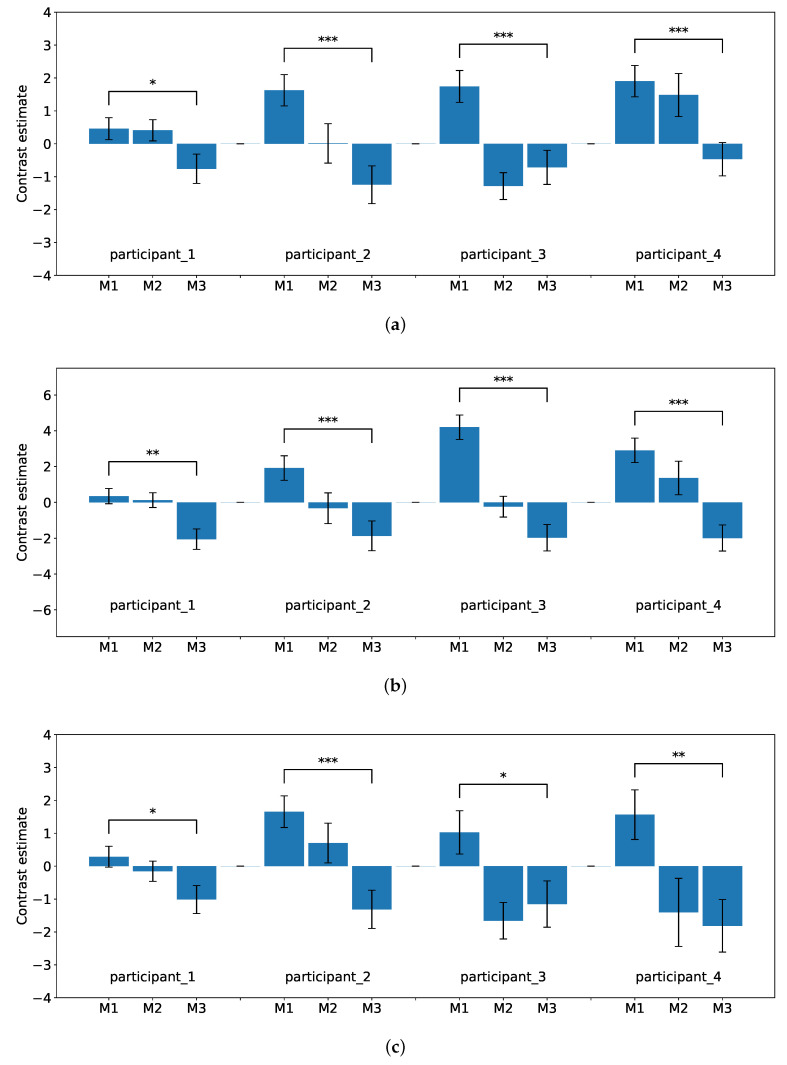

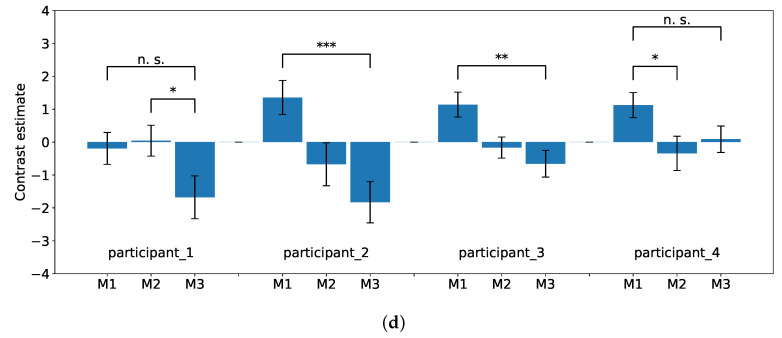
Trends in brain activities in different locations of the brain. For all plots, the Mean±SE is displayed. Asterisks indicate *p*-values (n.s. p>0.05, * p≤0.05, ** p≤0.01, *** p≤0.001 for the two-tailed Welch’s *t*-test). (**a**) Trends in brain activity in the premotor cortex.; (**b**) Trends in brain activity in the parietal lobe.; (**c**) Trends in brain activity in the dorsolateral prefrontal cortex.; (**d**) Trends in brain activity in the inferior frontal gyrus.

Moreover, investigations of differences in activation have focused on these regions in each participant. Table 1 shows the location of the brain regions with the maximum difference in activation between the first (M1) and third fMRI scans (M3) for each participant. Figure 8a–d show the degree of brain activity for each of the three tasks (M1, M2, and M3) in the experimental flow. The reports of brain activation were indicated with signal plots for the DLPFC, premotor cortex, parietal lobe, and inferior frontal gyrus. All signal plots show activity under the experimental condition (event type) relative to baseline (in arbitrary units [a.u.], ±90% confidence interval). The plots show activity patterns at the peak of activation (i.e., single voxel) as selected from the whole-brain contrast SPM map.

Performance data showed that each participant’s learning curve converged at the 100th and 150th tasks. The goal of this task was to observe only the brain activity related to executive function in each learning period. Reports have shown that executive function is closely associated with the parietal lobe and cerebellum, especially the PFC. The PFC has involved the establishment of target behaviors that are necessary for executive function. Environmental dependence is also said to be involved in goal maintenance. Moreover, conservation is involved in the flexible changing of goals.

Based on the results of the fMRI analysis, differences in activation were observed in the DLPFC (BA 9), parietal lobe (BA 7, 40), inferior frontal gyrus (BA 44), and premotor cortex (BA 6) in each participant. Comparing the brain activities during M1–M3, the differences followed decreasing trends. These brain regions are involved in the executive functions of complex behaviors, goal maintenance, flexible goal modification, and a combination of goals [14,15,16,17,18,19,33,34]. There have also been reports of decreases in related brain region activity as learning progresses [32,35,36].

Furthermore, the performance results in this experiment confirmed a convergence of the learning curve. We believe that the participants were in a state where they had completed sufficient learning and could perform efficiently.

In the fMRI data, brain regions involved in executive functions showed a decrease in brain activity after learning relative to before learning. This result suggests that the participants efficiently performed task anticipation, planning, and decision-making.

On the other hand, several other studies employing the ToH task have reported that the frontal pole is related to executive function [10,22]. However, the results of this experiment, which measured differences between pre-and post-learning, found no differences in the activation of this region.

This result suggests that there is not much effect on short-term learning for the frontal pole. Hence, long-time learning performed with trial and error increases brain activity.

## 7. Conclusions

In this study, the focus was on brain activity before and after learning. The effect of the planning and decision-making process concerning executive function on brain activity via long-time learning was investigated. The experimental design involved long-time learning in examining differences in cerebral activity. The participants performed individualized learning experiments involving the ToH to identify brain regions. As a result, brain regions involved in executive functions showed differences in activity between before and after learning.

This study focused on the DLPFC, parietal lobe, inferior frontal gyrus, and premotor cortex as brain regions involved in executive function. Activity in these brain regions declined compared with before learning. The results indicated a conflicting trend between learning progress and brain activity. Other studies on the relationship between learning progress and brain activity regions have reported this correlation [37,38,39,40,41,42,43]. Mulder et al. reported a firing correlation between single neurons and learning progress in an instrument learning experiment involving rats [37].

Studies using human shape identification tasks have also verified brain activity levels in trained and untrained states [38,39]. It has also been reported that there are some brain regions where the activity level is reduced in the trained state as compared with the untrained state.

The ability to solve the ToH requires task anticipation, planning, and decision-making. In this experiment, participant learning progressed, and their learning curves converged. Therefore, their problem-solving and task-anticipation abilities improved.

Moreover, the time required for the planning and decision-making process was simplified. It was also clear that the degree of activation tends to decrease with the number of tasks, such as through M2 to M3. The frontal pole, or the PFC, is related to the ToH task. Other studies have reported similar findings. For example, activation during the task was significantly higher than during rest. Further, differences have been reported between pre- and post-learning in short-term learning.

On the other hand, in the present long-time experiment, activation differences were obtained at the DLPFC, parietal lobe, inferior frontal gyrus, and premotor cortex after learning in each participant. We believe these activation differences were due to progressive learning about executive function.

This experiment was conducted for a long-time with only four participants due to logistic issues. Therefore, the conclusions driven by this research are not generic. More participants should be considered for sound and generalized conclusions. However, having more participants in this type of research is very challenging.

Nevertheless, we considered that the area of brain activation changes each time during long-time learning. This study analyzed behaviors and brain activities in M1–M3 in long-time learning. Exploring the dynamics of brain activity in long-time learning would also be essential. The learning curve for behavioral performance indicated this possibility.

Furthermore, a relationship with the dynamics of brain activities was observed. Various abilities are needed to solve the ToH task. The participants’ learning progress from learning curves was observed during the experiments. It can be stated that the observed learning progress implies improving overall solution ability. Therefore, the dynamics of brain activity, which are associated with this ability to contribute more, should be investigated in a future study. 

## Figures and Tables

**Table 1:** Peak coordinates with the most differences in activation.

Region Label	Participant No.	L/R	MNI Coordinates		
			x	y	z
Parietal lobe	1	L	−22	−10	72
	2	L	−4	6	66
	3	R	38	10	60
	4	L	−22	−2	66
Inferior frontal lobe	1	R	44	−70	48
	2	R	58	−24	14
	3	L	−26	−66	52
	4	L	−30	−50	62
Premotor cortex	1	L	−32	34	36
	2	L	−8	26	34
	3	L	−40	46	32
	4	L	−44	32	34
Dorsolateral prefrontal cortex	1	R	58	16	18
	2	L	−60	16	12
	3	R	46	16	14
	4	R	52	12	8

## Data Availability

Data will be available upon request.

## Abbreviations

The following abbreviations are used in this manuscript:

MDPI	Multidisciplinary Digital Publishing Institute
DOAJ	Directory of Open Access Journals
ToH	Tower of Hanoi
PFC	prefrontal cortex
DLPFC	dorsolateral prefrontal cortex
MHP	model human processor
fMRI	functional magnetic resonance imaging
MNI	Montreal Neurological Institute
